# The effects of Sutaehwan-Gami on menopausal symptoms induced by ovariectomy in rats

**DOI:** 10.1186/1472-6882-12-227

**Published:** 2012-11-23

**Authors:** Dong-Il Kim, Min Sun Choi, Sok Cheon Pak, Seung-bok Lee, Songhee Jeon

**Affiliations:** 1 Department of Obstetrics & Gynecology, College of Traditional Korean Medicine, Dongguk University, Gyeongju, Republic of Korea; 2 School of Biomedical Sciences, Charles Sturt University, Bathurst, NSW, 2795, Australia; 3 SungSung Traditional Korean Medicine Clinic, Yongdam-ro 115, Yeonsu-gu, Incheon 406-818, Republic of Korea; 4 Dongguk University Research Institute of Biotechnology, Dongguk University, 27-3, Phildong 3, , Joong-gu, Seoul, Korea 100-715

**Keywords:** Sutaehwan-Gami, Menopause, Ovariectomy, Uterus, Estrogen receptor, ERK, Akt

## Abstract

**Background:**

This study was undertaken to evaluate the beneficial effects of a modified prescription of Sutaehwan named Sutaehwan-Gami (SG), created by adding *Rhizoma dioscoreae* and *Carthami semen* to Sutaehwan, on menopausal symptoms.

**Methods:**

To evaluate the estrogenic effect of SG, we first examined estrogen receptor (ER) activation by SG treatment in breast adenocarcinoma cells and confirmed the estrogenic effect of SG *in vivo* ovariectomized rats. The animals were randomized into four groups: Sham operated group (Sham), saline treated ovariectomized group (OVX), SG treated group (SG) and raloxifene treated group (RLX). Animals were provided with SG at a dose of 500 mg/kg bw/day and RLX at a dose of 5.4 mg/kg bw/day with standard rat pellets for 3 months.

**Results:**

SG significantly increased ERα phosphorylation, and its downstream effectors, extracellular signal-regulated kinase (ERK) and protein kinase B (Akt) phosphorylation in breast adenocarcinoma cells. Treatment with SG reversed ovariectomy-induced uterine weight reduction and weight gain. Decreases in the levels of GOT and GPT were observed in the SG group. The significantly reduced E_2_β level in OVX rats was raised by treatment with SG. Moreover, SG significantly increased the phosphorylation levels of ERK and Akt in the uterus.

**Conclusion:**

Taken together, these data indicate that SG has phytoestrogen-like properties through ERK and Akt activation, implying that it could be protective and beneficial for the management of menopausal symptoms.

## Background

Menopause is an important physiological event, with the cessation of menstruation indicating the end of a woman’s reproductive lifespan. Women in menopausal transition experience a variety of symptoms such as hot flashes, sweating, anxiety, depression, mood swings, sleep disorders, vaginal dryness and joint pain; all of these symptoms are due to the cessation of ovarian estrogen production [1]. The major source of estrogen after menopause is through the conversion of adrenal androgen to estrogen by the enzyme aromatase in the peripheral tissues, but estrogen is maintained at a very low level due to aromatase inhibitors [2].

Estrogen plays multiple biological functions. It is required for the development and maintenance of reproductive tissues. Estrogen also controls energy homeostasis, core body temperature, bone remodeling and neuroprotection. Estrogen exerts its effects via the known estrogen receptors of ERα and ERβ by either nuclear- or membrane-mediated signaling mechanism depending on its target tissue [3]. Upon interaction with estrogen, receptors undergo structural changes to promote gene transcription. At the same time, estrogen triggers various protein kinase pathways including the MAPK/ERK and PI3K/Akt pathways [3]. Activation of these signaling cascades leads to the phosphorylation of the AF-1 domain of ERα [4].

Approximately 40% of women suffering such symptoms seek medical attention from health care providers for the management of menopausal symptoms [5]. Following the report from the Heart and Estrogen/progestin Replacement Study [6] that demonstrated an increased risk of cardiovascular disease and breast cancer among women randomized to hormone therapy and subsequent publication indicating a link between estrogen therapy and endometrial hyperplasia [7], many women refuse to use exogenous hormones and turn to alternative approaches for relief of menopausal symptoms. Herbal mixtures, in decoction or pill form, are one such alternative therapy, and the effects of decoction or pill can be shown after total and final reaction by their constituent compounds when administered to humans.

According to traditional Chinese medicine theory, both menopausal symptoms and threatened abortion are caused by kidney-liver weakness due to a deficiency of essence with accompanying yin-yang imbalance and organ disharmony [8]. Herbal formulas classified as kidney/liver-tonifying are thus considered suitable for the management of menopausal symptoms. Sutaehwan, which contains the four herbs *Semen cuscutae* (Dodder seed), *Ramulus taxilli* (Taxillus twig), *Radix dipsaci* (Himalayan teasel root) and *Colla corii asini* (Donkey-hide gelatin), has been used in Korea for the treatment of abortus habitualis including fetal restlessness in the uterus [9]. *Semen cuscutae* (Dodder seed) has been found to stimulate the reproductive system and reproductive endocrine function in male rats [10]. Moreover, it has shown a phytoestrogenic effect in MCF-7 cells [11]. *Ramulus taxilli* is traditionally used to tonify the kidneys and nourish the blood [9]. *Radix dipsaci* is proposed to enhance bone strength and has shown osteoprotective effect in ovariectomized rats [12]. *Colla corii asini* has long been used for tonifying the liver blood, arresting bleeding and supplementing the kidney yin fluid [9]. Since obesity is a common problem encountered in postmenopausal women as a result of reduced estrogen level [13], our study adopted a modified prescription, Sutaehwan-Gami (SG), that comprised sutaehwan with an addition of *Rhizoma dioscoreae* (yam), which has shown antiobesity and antihyperlipidemic activities [14,15], and *Carthami semen* (safflower seed) which is suggested to improve blood circulation to remove blood stasis and promote menstruation, and which exhibited lipid-lowering effects in humans with hyperlipidemia [16]. Our hypothesis is that Sutaehwan-Gami, consisting of six herbs, will strengthen kidney and liver function by nourishing the deficient essence in these organs, and will replace decreased estrogen and prevent the obesity related to menopause.

Our study aimed to determine whether SG has any estrogen-like properties since there have been no previous studies on the benefits of SG for menopausal syndrome. In the present study, ovariectomized rat was used as an animal model for menopause and we investigated the effect of SG on serum, body weight and uterus. We additionally evaluated the phosphorylation of ERα and ERK and Akt *in vivo* and *in vitro*.

## Methods

### Preparation of Sutaehwan-Gami

Sutaehwan-Gami, a dried decoction of a mixture of six herbs as listed in Table 1, was obtained from the College of Oriental Medicine of Dongguk University. A total of 64 g of herbs was added to 500 ml of water which was then boiled for 2 hr. The decoction was then filtered, lyophilized and stored at 4°C.

**Table 1:** **Prescription of Sutaewhan**-**Gami**

**Components**	**Part used**	**Amount (g)**
Semen Cuscutae	Seed	16
*Cuscuta chinensis *L.		
Ramulus Taxilli	Stem and branch	8
*Taxillus chinensis *De. (Loranthaceae)		
Radix Dipsaci	Root	8
Dried root of *Dipsacus asperoides*		
Colla Corii Asini	Donkey hide	8
*Equus asinus *L. (Equidae)		
Rhizoma Dioscoreae	Root	16
Rhizome of *Disoscorea opposita *Thunb.		
(Fam. Dioscoreaceae).		
Carthami Semen	Flower	8
*Carthamus tinctorius *L. (Asteraceae).		
Total amounts		64

### Cell culture

MCF-7 human breast cancer cells were purchased from the American Type Culture Collection (ATCC, Manassas, VA, USA). Cells were cultured in DMEM (WelGENE, Daegu, Korea) medium supplemented with 10% fetal bovine serum (WelGENE, Daegu, Korea), 100 unit/ml penicillin (WelGENE, Daegu, Korea) and 100 μg/ml streptomycin (WelGENE, Daegu, Korea). The cells were maintained in a humidified atmosphere containing 5% CO_2_ at 37°C. Cells were then treated with SG at 1 μg/ml for the indicated time (5, 10, 30, 60 and 120 min) to measure phosphorylation of ERα/ERK/Akt.

### Cytotoxicity assay

Cell viability was determined using 3-[4,5-dimethylthiazol-2-yl]-2,5-diphenyltetrazolium bromide (MTT, Sigma, USA) assay. In brief, MCF-7 cells were seeded on 24-well plates at a density of 5×10^5^ cells/well and treated with various concentrations (0.01, 0.1, 1.0, 5.0 and 10 μg/ml) of SG for 48 h. The medium was removed and the cells were incubated with 2 mg/ml of MTT solution. After incubation for 4 h at 37°C and 5% CO_2_, the supernatants were removed and dimethyl sulfoxide (DMSO, Sigma, USA) was added. The reactants were measured in terms of optical density (OD) at 590 nm with a microplate reader (UV max, Molecular Devices, USA). The optical densities were converted into percentages using the following formula:

Cell viability (%) = OD sample/OD negative control × 100. Negative control cells were treated with complete DMEM alone.

### Western blotting analysis

Thirty micrograms of cell lysates were electrophoresed in SDS-polyacrylamide gels (SDS-PAGE) and transferred to nitrocellulose membranes, which were then incubated with anti-phospho ERα (Ser118), anti-ERα, anti-phospho Akt (Thr308), anti-Akt, and anti-phospho ERK1/ERK2 (Thr202/Tyr204), anti-ERK1/ERK2 (Cell signaling, USA) antibodies for 16 h at 4°C. After washing with TBS-T (0.05%), the blots were incubated with horseradish peroxidase-conjugated anti-rabbit or anti-mou**s**e IgG, and the bands were visualized using the ECL system (Pierce Biotechnology, Rockford, IL, USA). Band images were obtained by using a Molecular Imager ChemiDoc XRS^+^ (Bio-Rad, Hercules, CA, USA) and band intensity was analyzed using Image Lab™ software version 2.0.1 (Bio-Rad).

### Experimental animals

Protocols for animal use were reviewed and approved by the Institutional Animal Care and Use Committee at the Dongguk University Ilsan Hospital (No. 2010–0530) and were in accordance with National Institute of Health guidelines. Healthy Wistar strain albino female rats (200-250 g, 8 weeks old) were obtained from OrientBio (Seoul, Korea) and were allowed 1 week for quarantine and acclimatization. Animals were housed under conditions of constant temperature (22±1°C), relative humidity (55±1%) and 12 h light/12 h dark cycle (light on at 7:00 am). They were housed in polycarbonate cages and given tap water and commercial rodent chow (Samyang Feed, Daejeon, Korea) *ad libitum*. The rats were blindly randomized into four groups, each group containing ten rats: sham operated group (Sham), saline treated ovariectomized group (OVX), Sutaehwan-Gami treated group (SG) and raloxifene treated group (RLX). We provided the animals with SG at a dose of 500 mg/kg bw/day and RLX at a dose of 5.4 mg/kg bw/day with standard rat pellets for 3 months. In the sham group, rats were operated but the ovaries were not removed. Treatment started 1 week after bilateral ovariectomy under anesthesia with 3% sodium pentobarbital. One day after 3 month treatment, the rats were anesthetized with i.p. injection of Rompun (0.04 ml/100 g) and Zoletil (0.04 ml/100 g). The animals were quickly dissected and cardiac blood, ovaries and uteri were collected for further analyses. Blood samples were allowed to clot at room temperature and the serum was separated by centrifuging at 400 g for 15 min. Body weight (BW) was recorded on the last day of the experiment. The uteri were removed and adhering fats were trimmed away. The uterine index (UI, %) was calculated by dividing the uterine weight by body weight. All specimens and serum samples were stored at −80°C until used for further assays.

### Laboratory measurement

Serum concentrations of total cholesterol (TC), high density lipoprotein (HDL) cholesterol, triglyceride (TG), glucose, glutamic oxaloacetic transaminase (GOT), glutamic pyruvic transaminase (GPT) were measured by enzymatic methods using commercial assay kit (Asan Pharm., Seoul, Korea). The calcium (Ca) and phosphorus (P) levels were measured by colorimetric assay kits (Biovision, USA). Alkaline phosphatase (ALP) and estradiol-17β (E_2_β) concentrations were determined using the BioVision assay kit (BioVision, USA) and Assay Design EIA kit (Assay Design, Ann Arbor, MI, USA), respectively. Nitric oxide (NO) is rapidly oxidized to nitrite and nitrate, which can be used to determine NO production. Nitric oxide colorimetric assay kit (Biovision, USA) was used to measure the total nitrate/nitrite in the samples. Measurement was carried out according to manufacturer protocol.

### Histological analysis

Uterine tissue specimens were fixed in 10% formalin for at least 24 h at room temperature. After fixation, tissues were dehydrated in graded ethanol, cleared in xylene, and embedded in paraffin. Thin sections (4 μm) were mounted on glass slides, dewaxed, rehydrated to distilled water, and stained with hematoxylin, and eosin. As part of the histological evaluation, all slides were examined by a pathologist, without knowledge of the previous treatment, under a light microscope.

### Data analysis

Results were given as mean ± S.E. or mean ± S.D. Data were analyzed using one-way analysis of variance (ANOVA) followed by Tukey’s *post hoc* test for multiple comparisons where appropriate. A p-value of 0.05 or less was considered as indicative of a significant difference.

## Results

### Effect of SG on cell viability

**Figure 1:**
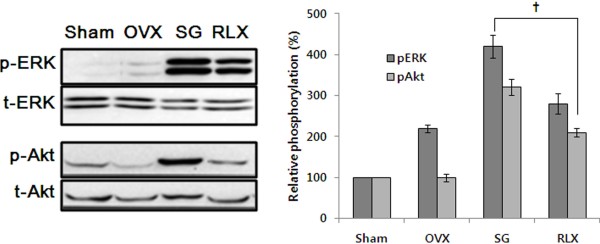
**Effect of SG on cell viability. **MCF-7 cells were treated with different concentrations of SG for 48 hours. Cell viability was determined by MTT assay. Data are mean ± SE of three experiments performed in triplicate. *P < 0.05 versus 0 μg/ml.

The potential cytotoxic effect of SG was investigated by determining its effect on the viability of a human breast adenocarcinoma cell line, MCF-7 cells. Cells were treated with SG at concentrations of 0.01, 0.1, 1.0, 5.0 and 10.0 μg/ml. After 48 h SG treatment, cell death was examined by MTT assay. SG showed no significant cytotoxicity by 5 μg/ml of SG but treatment with the highest dose (10.0 μg/ml) of SG reduced cell viability by 74.51 ± 7.1% (Figure 1).

### Effect of SG on phosphorylation of ERα/ERK/Akt in MCF-7 cells

**Figure 2:**
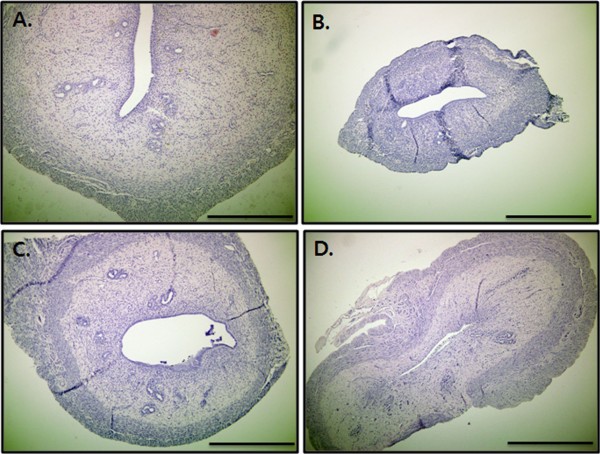
**Effect of SG on the activation of ER**α, **ERK and Akt in the MCF**-**7 cells. **Total and phosphorylation state of each protein was measured in MCF-7 cells which were treated with SG at 1 μg/ml for the indicated time. The whole cell lysates were immunoblotted with anti-p- ERα/ERK/Akt or ERα/ERK/Akt antibody. The intensity of the phosphorylated or total ERα/ERK/Akt was quantified by densitometry, and the intensity of the phosphorylated ERα/ERK/Akt band was normalized to that of total ERα/ERK/Akt. *P < 0.05 versus control.

In order to examine the estrogenic effect of SG, we measured phosphor-ERα and total-ERα from MCF-7 cells human breast adenocarcinoma cells in the presence of 1 μg/ml SG after different time durations. As shown in Figure 2, SG treatment led to an increased phosphorylation of ERα into the medium, which reached maximum at 120 min. Since phosphorylation of ERα occurs through the action of second messenger signaling pathways, the phosphorylation levels of ERK and Akt were measured. A dose of 1 μg/ml SG induced a rapid, 2-fold increase in the phosphorylation state of ERK at 5 min and then gradually increased to 60 min. Akt phosphorylation reached a maximum level at 30 min of SG treatment and this relatively high level of phosphorylation was maintained up to 2 hours.

### Body weight and uterine index in ovariectomized rats

The body weights of the ovariectomized and sham rats were not significantly different at the start of the study (data not shown). After three months, placebo-treated OVX rats weighed 22.4% more than sham rats (P < 0.05) despite a similar food intake (Table 2). Treatment with raloxifene but not with SG prevented the ovariectomy-induced weight gain (P < 0.05, versus OVX). Uterine index was significantly reduced in the OVX group (P < 0.01, versus sham group). Treatment of ovariectomized rats with either SG or raloxifene increased the uterine index compared with the OVX group, though not significantly.

**Table 2:** **Effects of SG on serum biochemical markers**, **body weight and uterine index in ovariectomized rats**

**Group**	**TC (mg/ ml)**	**HDL (mg/ ml)**	**TG (mg/ ml)**	**Gluc (mg/ ml)**	**GOT (Karmen/ml)**	**GPT (Karmen/ml)**	**Ca (μg/ml)**	**P (nmol/ml)**	**ALP (μmol/ml)**	**E2β (pg/ml)**	**NO (mM/ ml)**	**BW (g)**	**UI (%)**
**Sham**	**0**.**59 **± **0**.**01**	**0**.**20 **± **0**.**02**	**1**.**11 **± **0**.**05**	**2**.**38 **± **0**.**20**	**66**.**82 **± **2**.**68**	**17**.**68 **± **1**.**52**	**1**.**26 **± **0**.**05**	**5**.**80 **± **0**.**47**	**29**.**37 **± **2**.**828**	**484**.**83** ±**33**.**92**	**4**.**86 **± **0**.**43**	**260**.**00 **±**21**.**68**	**0**.**16 **±**0**.**02**
**OVX**	**1**.**07 **± **0**.05**	**0**.**23 **± **0**.**01**	**1**.**04 **± **0**.**14**	**2**.**55 **± **0**.**19**	**77**.**32 **± **4**.**66**	**36**.**63 **± **8**.**41**	**1**.**30 **± **0**.**22**	**6**.**64 **± **0**.**06**	**32**.**02 **± **6**.**11**	**296**.**30 **±**10**.80**	**4**.**88 **± **0**.**83**	**335**.**71 **±**18**.22*	**0**.**02 **± **0**.00**
**SG**	**1**.**08 **± **0**.**07**	**0**.**27 **± **0**.**02**	**0**.**41 **± **0**.**08**†	**3**.**16 **± **0**.**21**	**52**.**15 **± **2**.**42**†	**8**.**74 **± **2**.**69**†	**1**.**16 **± **0**.**07**	**7**.**16 **± **0**.**05**	**38**.**21 **± **6**.**72**	**430**.**68 **± **17**.**45**†	**4**.**18 **± **0**.**32**	**314**.**29 **± **33**.**10**	**0**.**08 **± **0**.**08**
**RLX**	**0**.**67 **± **0**.**04**†	**0**.**27 **± **0**.**05**	**0**.**26 **± **0**.**04**††	**3**.**37 **± **0**.**19**	**53**.**39 **± **3**.**99**†	**11**.**34 **± **1**.**63**†	**1**.**05 **± **0**.**10**†	**7**.**24 **± **0**.**08**	**42**.**68 **± **10**.**28**	**405**.**45 **± **15**.**36**†	**3**.**11 **± **0**.**28**	**257**.**14 **± **9**.**51**†	**0**.**07 **± **0**.**01**

Mean ± S.D. ** P < 0.01, * P < 0.05 vs sham, ^††^ P < 0.01, ^† ^P < 0.05 vs OVX.

### Serum biochemical markers in ovariectomized rats

The effects of SG on serum biochemical markers are summarized in Table 2. OVX rats showed significant increase of TC (P < 0.01, versus sham group), but raloxifene prevented ovariectomy-induced increase in TC (P < 0.05, versus OVX). Treatment with either SG or raloxifene significantly reduced the serum TG compared to the saline-treated OVX group. Ovariectomy appears to increase serum ALP, Ca and P levels, but these changes did not reach statistical significance. However, treatment of ovariectomized rats with raloxifene significantly altered serum Ca (P < 0.05, versus OVX). The effects of SG on liver were determined by the measurement of serum GOT and GPT levels. The OVX group exhibited an elevation in serum levels of GOT and GPT, indicating liver injury. Decreases in the levels of GOT and GPT were observed in the SG and raloxifene groups (P < 0.05, versus OVX). As expected, the significantly reduced E_2_β level in saline-treated ovariectomized rats (P < 0.01, versus sham group) was raised by treatment with either SG or raloxifene (P < 0.05, versus OVX). NO appears to be low with the treatment with either SG or raloxifene.

### Effect of SG on phosphorylation of ERK and Akt in the uterus of ovariectomized rats

**Figure 3:**
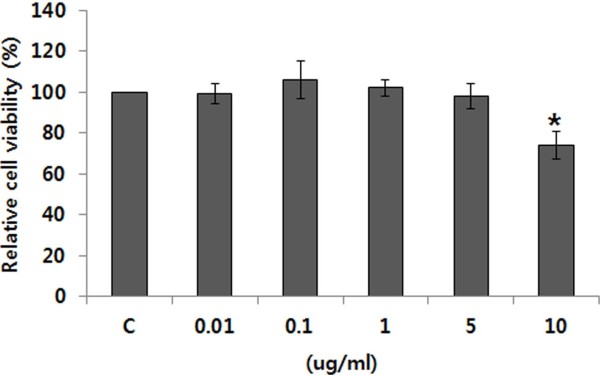
**Effect of SG on the activation of ERK and Akt. **Ovariectomized rats were treated with SG or raloxifene for 3 months. The uterine tissue lysates were immunoblotted with anti-p-ERK, anti p-Akt, ERK antibody and Akt antibody. The intensity of protein was quantified by densitometry and the intensity of the phosphorylated ERK or Akt band was normalized to that of total ERK or total Akt, respectively. †P < 0.05 versus OVX.

Our study showed that ovariectomy led to an increase of phosphorylation of ERK. To investigate the long-term effect of a herbal mixture with estrogen-like properties on activation of ERK and Akt *in vivo*, we treated ovariectomized rats with SG for 3 months. It was found that SG and raloxifene increased the phosphorylation levels of ERK and Akt in the uterus (Figure 3).

### Uterine morphology in ovariectomized rats

**Figure 4:**
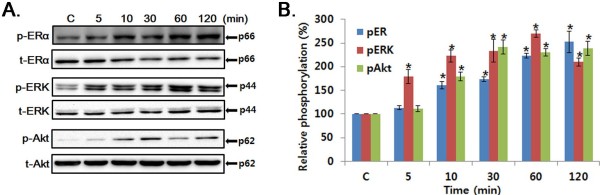
**Effect of SG on uterine morphology in ovariectomized rats. **Following three month treatment, animals were sacrificed and uterus was removed. Formalin-fixed rat uteri were paraffin-sectioned serially into 4 μm sections to be stained with hematoxylin and eosin. **A**: Sham, **B**: OVX, **C**: SG, **D**: RLX, Bar = 500 μm.

Under light microscopy, a marked atrophy of the uterus was observed in ovariectomized rats that was about half the size of that in sham rats (Figure 4). Even though our study did not measure the uterine histologic parameters, this finding might be due to decreased epithelial cell height and myometrial thickness. A partial reversal of uterine atrophy was seen in ovariectomized rats treated with either SG or raloxifene, probably due to an increase in the amount of collagen in the stroma.

## Discussion

Ovariectomy is a standard surgical procedure to induce menopause in experimental animals and ovariectomized female rats show a dramatic cessation of ovarian function and higher risk of osteoporosis [17]. Our study showed that those rats which were ovariectomized and not treated with either SG or raloxifene showed a significant decrease in uterine weight compared to those rats which underwent only sham operation. The reduction of uterine weight was due to an atrophy of endometrium resulting from a lack of hormones secreted by the ovaries. Black *et al*. [18] have described further evidence of reduced uterine weight by the reduction of uterine epithelial height, uterine myometrial thickness and uterine stromal expansion. Recent data show that ovariectomy also caused atrophy of the vaginal epithelium in rats [19]. However the administration of SG to ovariectomized rats for three months appeared to increase the weight of the uterus, mainly due to lack of uterine atrophy compared to the placebo-treated ovariectomized rats. This improvement in uterine atrophy is probably due to the presence of biologically active phytoestrogen-like molecules in SG. It is interesting to note that the insignificant change of uterine weight in raloxifene-treated ovariectomized rats seen in our study confirms the fact that raloxifene lacks uterotrophic activity due to its anti-estrogenic properties in the uterus [18].

Our study showed ovariectomized rats significantly increased body weight, which was also observed in other studies [19,20]. The effect of estrogen insufficiency on lipid metabolism during the menopause has been well documented since postmenopausal women receiving estrogen replacement therapy have been found not to display the characteristic pattern of body weight and BMI increase including a shift to a central android fat distribution associated with menopause [21-23]. Furthermore, aromatase-knockout mice displayed progressive accumulation of intra-abdominal adipose tissue compared with wild type counterparts in both males and females [24,25] demonstrating an important role for estrogen in the maintenance of lipid homeostasis. It is thus possible that some of the phytoestrogen-like molecules in SG were involved in the regulation of lipid metabolism although the prevention of ovariectomy-induced weight gain by SG was not significant. The magnitude of the lipid metabolism effect of raloxifene was more distinguishable than that of SG.

The serum biochemical profiles in our SG- or raloxifene-treated rats showed mixed findings. SG and raloxifene both produced a marked reduction in serum triglyceride concentrations. Since the administration of estrogen to postmenopausal women increased triglyceride levels [26] and changes in triglyceride levels were negatively associated with changes in estradiol levels in premenopausal women [27], it follows that changes in triglyceride concentrations in our study must be related with changes in body weight. A trend to a progressive increase in body weight during menopausal transition is closely associated with an increase in total cholesterol and LDL cholesterol levels, and no change in HDL cholesterol levels [27]. Our ovariectomized rats showed no significant change in HDL concentrations. Thus loss of female steroid hormone alone did not influence HDL cholesterol level. While the hypocholesterolemic effect of raloxifene was obvious, SG showed minimal lowering of cholesterol.

Another characteristic of menopausal symptoms is bone loss. The measurement of ALP activity can be used as a barometer of bone formation and bone resorption *in vivo*. It is common to see an increase in serum ALP activity and subsequent DPD (deoxypyridinoline, a breakdown product of collagen during bone resorption) level in response to ovariectomy [28]. Our results indicate that treatment with SG did not lower serum ALP activity, suggesting that SG might not have enough potent phytoestrogen-like molecules to affect the bone turnover rate in rats.

Menopause is further associated with increased oxidative stress and metabolic disorders which can cause several age-related diseases [17]. In fact, estrogen has potent antioxidant effects and can reduce risk factors for general cardiovascular diseases by reducing inflammation [29]. Behr *et al*. [17] confirmed that ovariectomized rats presented increased plasma oxidative stress. NO controls the vascular tone and blood flow by activating soluble guanylate cyclase in the vascular smooth muscles. Abnormalities in vascular NO production thus result in endothelial dysfunction [30]. The discrepancy between NO and estradiol profiles from SG administration implies that the estrogenic effects of SG may not be mediated by an increase production of NO in our study. Different oxidative damage parameters need to be analyzed by further studies to clarify this discrepancy. It is true that the measurement of NO is problematic due to its short half-life (~ 5 s) and its high reactivity with other biological components [31].

Phosphorylation of ERα is enhanced in response to estrogen binding and through the action of second messenger signaling pathway [32]. ERK and Akt are serine/threonine protein kinase, which plays a major role in mitogenic signaling. Here we report that SG-induced ERα phosphorylation occurs within minutes. The *in vitro* phosphorylation pattern of ERK and Akt was similar to that of ERα. In our *in vivo* data, rat uterus under the influence of SG similarly mediated the phosphorylation of ERK and Akt. These results lead to a model in which the posttranslational modification of protein kinase cascades is the result of a transcription-independent and nongenomic action of estrogen receptor.

## Conclusion

In conclusion, SG as a dried decoction consisting of six herbal medicines was proven to have phytoestrogen-like properties in female ovariectomized rats. The data presented here increase our knowledge about the protective and beneficial mechanisms of SG for menopausal symptoms, and it may therefore be a viable candidate compound for the development of therapeutic drugs for the management of menopausal symptoms.

## Abbreviations

SG: Sutaehwan-gami; DPD: Deoxypyridinoline; TC: Total cholesterol; HDL: High density lipoprotein; TG: Triglyceride; GOT: Glutamic oxaloacetic transaminase; GPT: Glutamic pyruvic transaminase; ALP: Alkaline phosphatase; E_2_β: Estradiol-17β; BMI: Body mass index.

## Competing interest

All authors manifest that there is no conflict of interests.

## Authors’ contributions

SB carried out the animal experiments, namely ovariectomy and dissection of uterus. SH carried out in vitro cellular experiments and immunoblotting. SC participated in the design of the study and performed the statistical analysis and wrote the draft manuscript. MS carried out the revised experiment in MCF-7 cells. DI conceived of the study, and participated in its design and coordination. All authors read and approved the final manuscript.

## Pre-publication history

The pre-publication history for this paper can be accessed here:

http://www.biomedcentral.com/1472-6882/12/227/prepub
